# Factors influencing access and utilization of health services among older people during the COVID − 19 pandemic: a scoping review

**DOI:** 10.1186/s13690-021-00719-9

**Published:** 2021-11-07

**Authors:** Peivand Bastani, Mohammadtaghi Mohammadpour, Mahnaz Samadbeik, Misagh Bastani, Giampiero Rossi-Fedele, Madhan Balasubramanian

**Affiliations:** 1 Health Human Resources Research Center, School of Management and Medical Information Sciences, Shiraz University of Medical Sciences, Shiraz, Iran; 2 Social Determinants of Health Research Center, Yasuj University of Medical Sciences, Yasuj, Iran; 3 Social Determinants of Health Research Center, Lorestan University of Medical Sciences, Khorramabad, Iran; 4 Anesthesiologist, Shiraz University of Medical Sciences, Shiraz, Iran; 5 Adelaide Dental School, The University of Adelaide, Adelaide, SA 5000 Australia; 6 Research Fellow and Lecturer, Faculty of Medicine and Health, The University of Sydney, Sydney, Australia

**Keywords:** Access, Utilization, Healthcare services, Elderly, COVID-19

## Abstract

**Background:**

Access to healthcare and service utilization are both considered essential factors for improving the general health and wellbeing of older people, especially at the time of COVID-19 pandemic. The aim of the study is to explore factors affecting healthcare access and health service utilization for older people during the pandemic.

**Methods:**

PubMed, Web of Science, Scopus and Embase were systematically searched for relevant articles. Access, utilization, health, elderly and COVID-19 were used as keywords in the search strategy. A total of 4308 articles were identified through the initial database search; 50 articles were included in the review as passing the eligibility criteria. The searches were conducted up to August 2021. Data extraction was performed, and evidence was descriptively illustrated. Thematic analysis was used to explore factors influencing the elderly’s access and utilization of healthcare services, using Max QDA_10_, a qualitative analysis software.

**Results:**

Among articles included in the review (*n* = 50), a majority of the studies were from the United States (36%), followed by India (8%). According to the main healthcare services, a large number of articles (18%) were related to mental health services, followed by digital health services (16%). Factors were identified at an individual, provider and systems level. Seven main themes emerged from the thematic analysis, as determinants of elderly’s access and utilization of healthcare services during COVID-19 pandemic. These included: access to non-COVID related services, access to COVID-related services, literacy and education, accommodation challenges, perceived attitudes of aging, and policies and structures, and social determinants.

**Conclusion:**

Mental health and digital health services were identified as major issues influencing or contributing to or influencing older people’s health during the COVID-19 pandemic. We also argue on the importance of a rounded view, as attention to a range of factors is vital for policy decisions towards sustainable care and equitable interventions for improving the health of older people.

**Supplementary Information:**

The online version contains supplementary material available at 10.1186/s13690-021-00719-9.

## Background

Sustainable access and improved utilization to healthcare services are vital towards the physical, social, and mental health and of older people [1]. Several studies have identified that psychological, physical and economic barriers can influence health care access among the older population groups [2]. In pandemic scenarios, such as COVID-19, these barriers to access and utilization of services can appear prominent. Germain and Yong (2020) suggest that some barriers can appear amplified, contributing to further inequalities to access to health services during the COVID-19 pandemic. These barriers include differences in perception to medical issues among various ethnic groups, cultural issues, gender, information barriers, legal barriers and the stigma related to the disease [3].

In general, older people encounter more barriers to access and utilization, when compared with other groups due to a number of factors ranging from their physical conditions to disabilities and mental problems. Radwan et al. (2020) have mentioned five main challenges that older people experience during COVID-19 pandemic [4]. These include: violence, misinformation, nutritional challenges, problems related to wellbeing, and limitations to routine activities [4]. According to Neumann-Podczaska et al. (2020) a large number of symptoms may occur following the onset of COVID-19 in old people. These symptoms include failure and complications associated with respiratory, gastrointestinal, cardiovascular, and neurological systems [5]. All the aforementioned, can highly worsen the condition and make it more complicated for the older people.

To date, there is growing evidence on issues of access and utilization of health services for older people during the pandemic. Due to the growing importance of the social determinants influencing older people care and costs involved in the health system [6], understanding access and utilization to necessary services during the pandemic is vital. A better understanding of these impacts can be significant for the health policymakers and the health care providers for better planning and provision of inpatient services, Intensive Care Units (ICUs) and hospitalizations and outpatient and routine care services for older people. Therefore, the present study aims to explore the factors affecting the elderly’s access and utilization to health services during the pandemic through a comprehensive investigation of available literature mainly to support health planners, providers and policymakers.

## Methods

Scoping review methods were adopted for this study. Broadly, scoping reviews attempt to initially assess the scope of those available evidence to determine the nature and conceptual boundaries of the topic [7]. According to Joanna Briggs Institute’s, scoping reviews bring potential in mapping the key concepts of the research along with also making the definitions and the concepts more explicit [8]. In this review, the approach proposed by Levac, Colquhoun and ‘O’Brien (2010) [9] was applied. This approach included six main steps for conducting the scoping reviews as follows:

I. **Clarifying and linking the purpose and research question**
  This scoping review was designed to answer the question that "what are the main factors affecting the access and utilization of health services among the elderly population during COVID -19 pandemic?"
II. **Balancing feasibility with breadth and comprehensiveness of the scoping process**
  The main keywords were agreed by the research team considering the research question. The main keywords include utilization, access, health, elderly and COVID-19. For achieving more sensitivity, the logical operators OR /AND were used to combine the related keywords. Four main databases of PubMed, Scopus, Web of Science, and Embase were searched systematically according to the search strategy (Table 1). The scoping review was conducted in February and March 2021. Data was searched and updated up to 08.14.2021.

**Table 1:** Search strategy for the scoping review

PubMed	Aged [Mesh] AND (utilization [Title/Abstract] OR access [Title/Abstract] OR accessibility [Title/Abstract] OR “use of services”[Title/Abstract]) AND COVID-19[Mesh]
**Scopus**	(TITLE-ABS-KEY (“COVID-19” OR “coronavirus disease-19” OR “2019-nCoV” OR ncov OR “2019 novel coronavirus” OR “novel coronavirus” OR “acute respiratory syndrome coronavirus 2” OR sars-cov-2) AND TITLE-ABS-KEY (aged OR elder OR “old age” OR ageing OR elderly) AND TITLE-ABS-KEY (utilization OR access OR accessibility OR “use of services”)) AND (LIMIT-TO (LANGUAGE, “English”))
**Web of Science**	TOPIC: (aged OR elder OR “old age” OR ageing OR elderly) AND TOPIC: (utilization OR access OR accessibility OR “use of services”) AND TOPIC: (“COVID-19” OR “coronavirus disease-19” OR “2019-nCoV” OR ncov OR “2019 novel coronavirus” OR “novel coronavirus” OR “acute respiratory syndrome coronavirus 2” OR sars-cov-2)
**EMBASE**	)aged:ab,ti OR elder:ab,ti OR ‘old age’:ab,ti OR ageing:ab,ti OR elderly:ab,ti) AND (utilization:ab,ti OR access:ab,ti OR accessibility:ab,ti OR ‘use of services’) AND (“COVID-19”:ab,ti OR “coronavirus disease-19”:ab,ti OR “2019-nCoV”:ab,ti OR ncov:ab,ti OR “2019 novel coronavirus”:ab,ti OR “novel coronavirus”:ab,ti OR “acute respiratory syndrome coronavirus 2”:ab,ti OR sars-cov-2:ab,ti)

**Fig. 1:**
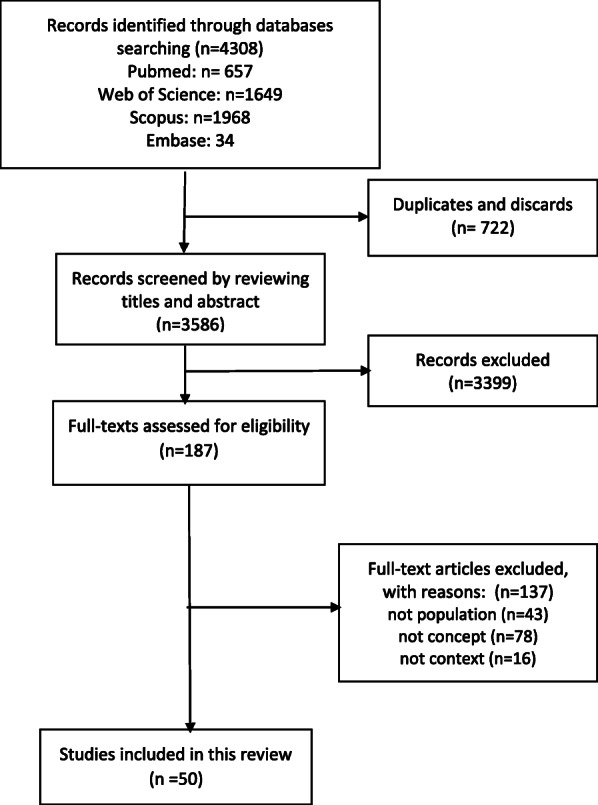
The PRISMA flowchart of the scoping review

III. **Using an iterative team approach to selecting studies and extracting data**
  Following the systematic search, applying the aforementioned search strategy, a total of 4308 articles were retrieved from the four databases. After refining for duplicates, 722 cases were eliminated. Title/abstract screening led us to *n* = 187 articles, which were included for full-text screening. Non-English articles, including conference proceedings and books were excluded. In general, a PCC (population, concept and context) was used as eligibility criteria along with the scoping review’s research question to screen the articles and include the most relevant ones. The present PCC was defined as follows: Population: elderly population, Context: access and utilization during COVID-19 pandemic and Concept: health service access and utilization. Articles that did not meet the PCC criteria and were not in line with the aims of the study were also excluded. According to this, the exclusion criteria was those articles with no full texts in English, articles with conference proceedings designs, books and book chapters and those articles which ‘weren’t coherent with the defined PCC. Endnote X7.1, by Thomson Reuters, was used as a reference manager software. The PRISMA flowchart that illustrates the aforementioned process is presented in Fig. 1.

**Fig. 2:**
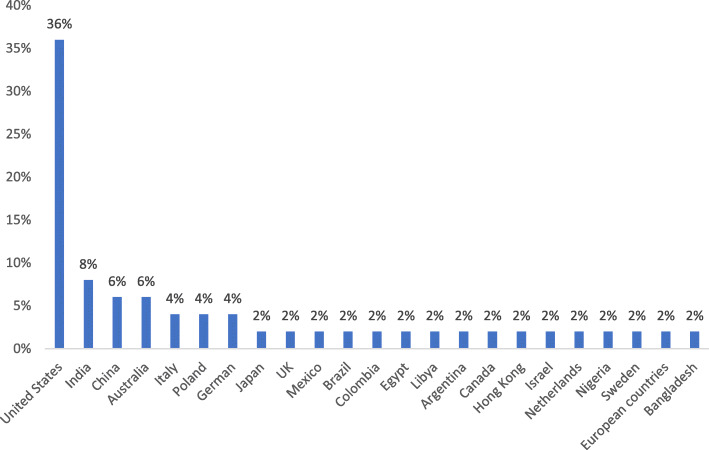
The frequency of the included articles according to the place of study

IV. **Incorporating a numerical summary and qualitative thematic analysis**
  The data extraction form was designed in Microsoft Excel 2013 containing the first authors` name, the year and place of study, types of healthcare services, the study aim and design, as well as the main results of the included studies (Table A-supplement). Data extraction was completed by two of the authors (MM and MS), and at times of disagreement, the third research member (PB) made the final decision. and the descriptive results of this step is presented in Fig. 2. A thematic analysis approach was applied for evidence synthesis.
V. **Identifying the implications of the study findings for policy, practice or research**
  In order to conduct the thematic analysis, Thomas and Harden’s approach was used [10]. This approach helped us to achieve and explore the content as the main and sub-determinants of access and utilization of health services by the elderly population during COVID-19 pandemic. To this purpose, after familiarization with the extracted data via continuous and mutual reviewing of the content, the research team tried to seek meaningful units according to the research question and created and labelled the initial codes. The process of open coding was continued and through a final reviewing and refining initial codes via merging the similar codes and omitting the duplications, the final codes were explored and labelled. Then the emergent sub-themes and the main themes were categorized into final codes. More than the themes` labels, in this step, the definitions and descriptions of the themes were considered, and the main and the sub-themes were tabulated (Table 2). In order to conduct the data analysis, we utilized a Qualitative Software for Data Analysis (MAX QDA) version 10.
VI. **Adopting consultation as a required component of scoping study methodology**
  Finally, in the last step of the scoping review, a conceptual thematic map was proposed for better illustration of the concepts and better understanding of health care policymakers and decision makers. The research team based on the explored themes and the sub-themes designed the initial draft of the thematic map that was finalized and confirmed by a mini panel of experts in the area of elderly health and public health.

**Table 2:** Main themes and sub-themes affecting the elderly’s access and utilization of healthcare services during COVID-19 pandemic

Main themes	Sub-themes	References
Access to COVID-related services	Acute COVID services	[5, 11, 12]
	Supplementary oxygen services	[5]
	ICU services	[13]
Access to non-COVID related services	Homecare	[14–16]
	Tele health	[17–28]
	Routine/Outpatient health care	[16, 21, 24, 27, 29–31]
	Oral healthcare	[24]
	Mental healthcare	[11, 12, 19, 29, 30, 32–38]
	Medications	[20]
	Palliative care services	[13, 23, 39]
	Chronic healthcare services	[40, 41]
	Other primary healthcare services	[42]
Literacy and education	Digital literacy	[19, 22, 28, 32, 34, 43]
	Misinformation	[11, 19]
	Continuing education	[35]
Accommodation challenges	Caregivers and nurses	[16, 17, 26, 39]
	Nursing homes	[15, 44, 45]
	Nutritional challenges	[29]
	Social support services	[46]
Policies and structures	Continuity of essential health services	[18, 47]
	Health policy priorities	[13, 43, 48]
	Organizational communication	[18, 44]
	Ethics during hospitalization	[49]
Perceived attitudes of aging	Uncertainty about the future	[29]
	Compliance to recommendations	[36]
	Reframing aging initiative	[48]
	Comprehensive understanding of ageism	[48]
Social determinants of health	Physical determinants	[5, 6, 17, 32, 43, 44]
	Economic determinants	[6, 29, 47, 50]
	Social determinants	[6, 11, 30, 32–34, 40, 44, 47, 50–53]
	Demographic determinants	[6, 30, 40, 42, 51, 53]
	Cultural determinants	[18]

## Results

A total of 4308 articles were available after a preliminary search from the four databases; 187 articles were included for full text reading, and a total of *n* = 50 articles were selected for this review after confirming eligibility. The majority of published literature came from the United States (*n* = 18; 36%), followed by India (*n* = 4; 8%). Figure 2 provides an illustration of the frequency of the included articles, based on the location the study was conducted (Fig. 2).

**Fig. 3:**
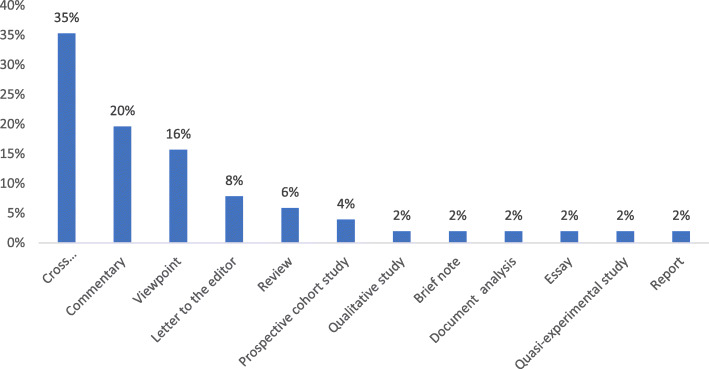
The frequency of the included articles according to types of healthcare services

A large number of studies were cross-sectional, followed by commentaries and viewpoint articles. (Fig. 3).

**Fig. 4:**
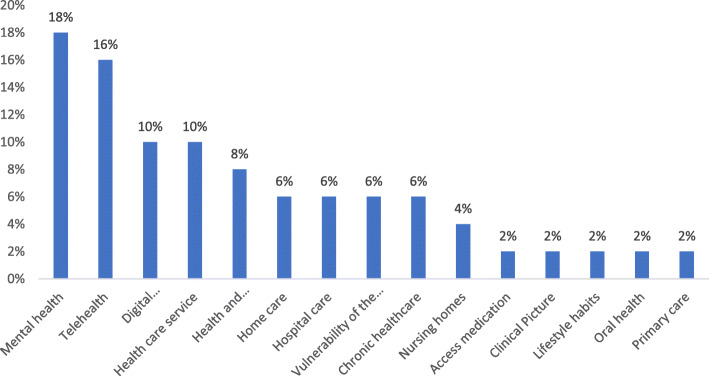
The frequency of the included articles according to the study type

According to the main healthcare services, most of the articles (9 articles; 18%) were related to mental health services, followed by Telehealth and digital health services (Fig. 4).

The thematic analysis led to seven main themes as follows: Social determinants, access to non-COVID related services, access to COVID-related services, literacy and education, accommodation challenges, perceived attitudes of aging, and policies and structures. These main themes are the sub themes of access and utilization of healthcare services among the elderly during COVID-19 pandemic is provided in Table 2. Further description and definition of the main themes and sub-themes are below.

**Fig. 5:**
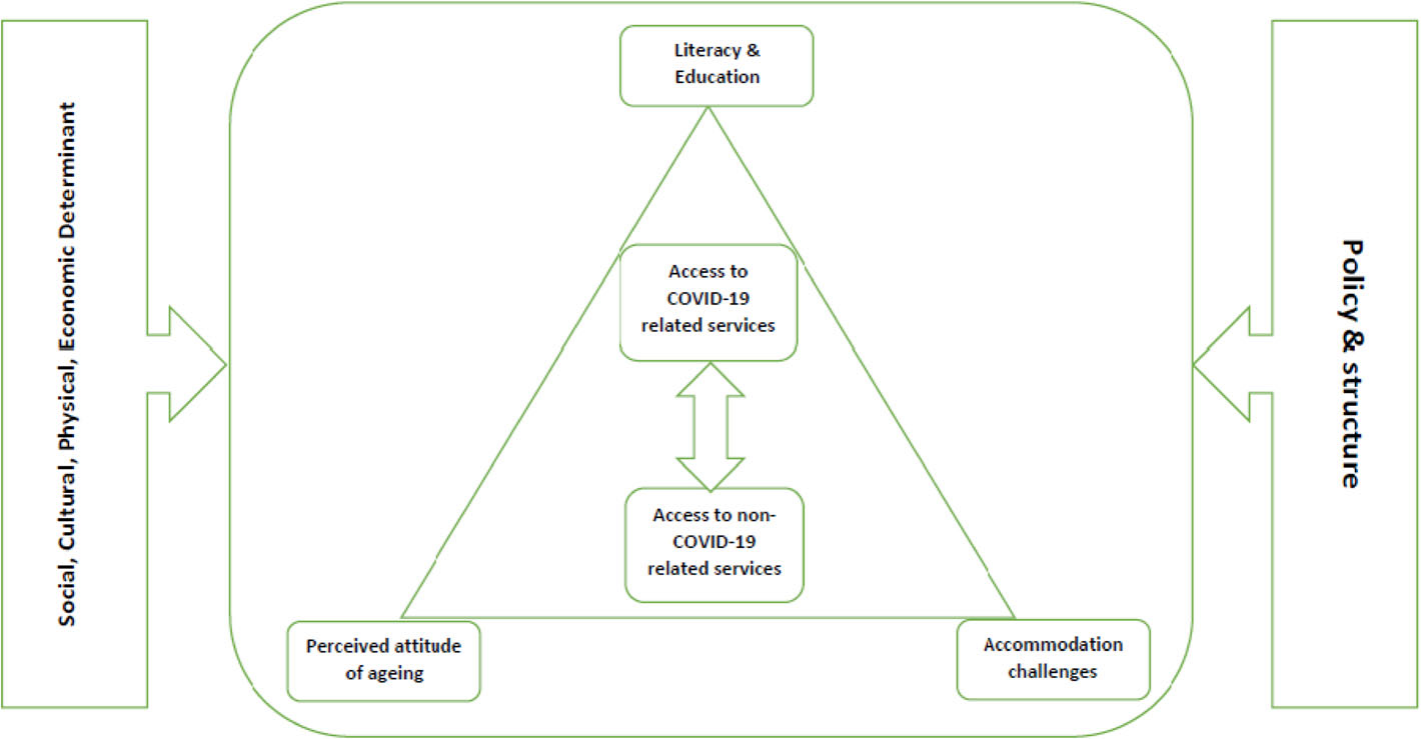
A thematic map of the scoping review

I. ***Access to COVID-related services***
  Synthesis of findings identified that the elderly population require specialized services due to a higher probability of morbidity among the population group. The subthemes accommodated three concepts: acute COVID-19 services, the need for supplementary oxygen and ICU services. According to the data from studies contributing to this theme, there is a higher occurrence of acute respiratory distress syndrome [12] and a high risk of presenting complications from COVID-19 [11] and the particular need for ICU service [54]. are among the main aspects. Also, contextual factors and underlying conditions of older people can determine the health needs and utilization of the health services during COVID-19 pandemic.
II. ***Access to non-COVID related services***
  Older people require access to non-COVID related services during the pandemic. Based on the studies' data, these services are presented as nine sub-themes of access to homecare, tele health services, routine/outpatient healthcare services, oral, mental and palliative services, medications, and chronic and other primary healthcare services.
  Studies have identified a higher prevalence of known risk factors for suicide [30, 33], increased risks of mental and physical health problems [32], susceptibility to the effects of stress and major depression [12, 29], probability of mental disorders [12] as well as preexisting or experience of loneliness [19, 30, 33, 35]. In addition, among the older people during the COVID-19 pandemic, the need to improving positive coping strategies [33] and more substantial psychosocial support [11] are considered as mental health strategies.
  In regard to oral health services, Leon et al. [24] have noted inequities in oral health care and dental services during COVID-19 for older patients. Palliative care as critical services for the elderly – studies have raised interdisciplinary palliative care approaches [23, 39], accompanied by the digital provision of such care [23].
  A further sub-theme is access to routine healthcare services. During COVID-19 pandemic, the number of physician consultations seems to have decreased [21] and some concerns about the maintenance of routine care of the older patients have been raised [21]. Limited access to routine health care [10] and reduced accessibility of health care for older patients [24, 30, 35] has potentially contributed to an increase in the number of delayed or missed medical appointments [30] and medical comorbidities [30, 35] among the elderly.
  Studies also raised the importance of medication delivery services, particularly on the establishment of medication impress systems [20]. Access to home care services is among other sub-themes in this area. Shortage of physicians for home visits and the restricted facilities for laboratory tests [15] can potentially affect the access to medical home visits [14] among the old population during COVID-19 pandemic.
  Tele health services have emerged as an important service during COVID-19. Studies have pointed to developing telehealth for old patients [11, 29, 33, 35, 39] while the others have mentioned new ways to use telehealth services similar to video visits [18], digital image prescriptions [20], E-Prescribing, online health services [22], tele palliative care [23] and teledentistry [24].
III. ***Literacy and education***
  Literacy and education of the older people also seemed to affect their access and utilization of the health services during the COVID-19 pandemic. Creating a continuous learning environment for older people and improving their digital literacy is vital. Implementing digital literacy programs in elderly populations [32] is emphasized in the included studies. At the same time, it shouldn't be forgotten that in such a population, there is always a potential for social and digital exclusion [22]. In other words, the use of virtual social media and other digital applications by old people can be accompanied by inconvenience, stress, incapability or not being user-friendy.
  More than improving the level of education, health literacy and digital literacy, another considerable sub-theme is the existence and development of misinformation [11, 19]. It should be noticed that false information can be disseminated very fast and with the higher speed and impact of accurate health information and education.
IV. ***Perceived attitudes of aging***
  As the process of aging occurs, the physical, mental and even social capabilities of the people are restricted. In this regard, it is essential for an old person to accept the situation and have a positive attitude. During the pandemic, this condition is intensified because of the uncertainty about the future [29]. Such a condition causes the necessity of more compliance to recommendations [36] along with a comprehensive understanding of ageism [48]. Reframing the aging initiative is among other strategies and solutions that can help increase access to health services and cope with the new condition by an old person.
V. ***Accommodation challenges***
  Another theme explored in this study is the challenges related to the accommodation of the old populations. This accommodation can include various types of nursing homes [15, 44, 45] for old people. However, this kind of accommodation can barely affect and worsen the condition of morbidity of the diseases, particularly during the COVID-19 pandemic. The elderly's nutritional challenges [29] and the issues related to their caregivers and nurses are also among the other sub-themes mentioned in this area.
VI. ***Policies and structures***
  More than the themes above that most of them have a direction toward the old patient or their required health services or conditions, the health policies and structures of the health systems can also be effective on the access to the services. Health policy priorities, the same as the development of the Age-Friendly University (AFU) Movement [48] and engaging in policy change through investments in social protection [43], are among what was mentioned in the included evidence. Another important sub-theme in this area is the existing policies and structure to preserve the continuity of critical health services during the COVID-19 pandemic. Multidisciplinary approaches [18] can be helpful in this regard. At the same time, the policymakers should be aware of the negative impacts of decreasing the demand and supply for non-COVID-19 healthcare services [47] that can directly threaten the continuity of the services for old people.
  About the other sub-theme in this area, organizational communication, the included evidence have emphasized the need to create a link with local community-based organizations [18] and attention to the local government–based support programs for community-dwelling older adults [44]. And finally, the ethical dilemma in care for the elderly during hospitalization [49] is the last issue requiring consideration to improve the access of the old population to health services during COVID-19 pandemic.
VII. ***Socio-cultural***
  Social, cultural, economic, and physical determinants can affect access and utilization of health services on a large scale. Demographic determinants, the same as gender [6, 30, 51] and the old person's marital status [6], can directly affect access to the required health services. Physical determinants like the old person's physical immobility [6], his/her perception of self-health [32] and the increased risks of mental and physical health problems [44] are among the most significant related items in the included evidence. Cultural determinants, the same as cultural, social and language factors [18] are also can be effective in the access and utilization of health services among the elderly.
  Social determinants include a wide range of factors, according to the included evidence. For instance, 'place of residence [6, 50], their social group [6, 51] and group activities [34], limited social activities [11] and social networks [11] accompanied 'with living arrangements [6] and the inequalities related to rural/urban inhabitants [50] and being homebound [52] are among the essential social determinants in the present literature. Moreover, the extensive social networks that can be accessed by the elderly was among an essential item in this regard [53].
  And finally, the economic determinants are the last sub-theme in this area. The included evidence have noticed the economic levels of the old population [6, 50], their financial resources [50] and also financial concerns [29]. The elderly's economic dependence [6, 50] can also be noticeable as an effective factor on the access and utilization of health services.
  Finally, for a better illustration of the main themes and creating a map for policymakers and health managers, a thematic map of the scoping review is presented (Fig. 5).

According to Fig. 5, the mutual relationship between the access of the elderly to COVID-19 related services and non COVID-19 related is centrally mentioned that can be affected by the personal determinants the same as the elderly’s accommodation challenges and their perceived attitude of aging. At the same time, the determinant of literacy and education can have the same role at this level. It should not be forgotten that micro determinants are not the only factors that can affect the elderly’s access and utilization to health services during the pandemic, but also macro determinants. Health policies and the system’s structure, along with complex demographic, physical, social, cultural and economic factors, can also play a dominant role in this regard.

## Discussion

Results of the present scoping review have shown that access to non-COVID related services, access to COVID-related services, literacy and education, accommodation challenges, perceived attitudes of aging, and policies and structures can influence the access and utilization of healthcare services among older people during the COVID-19 pandemic. According to the Build Back Fairer: The COVID-19 Marmot Review, the COVID-19 condition has intensified the inequalities in health among the whole community. According to the review, the risk of mortality rate because of the disease has increased due to socio-economic and ethnic inequality among the populations. For instance, a higher rate of death has been reported among the homeless population, those living in deprived areas or overcrowded shelters, those who work closely with others, those with poorer health conditions, and the elderly population [55].

As the elderly are considered as a vulnerable group due to their physical, mental and social conditions and their economic status, the results of the present study highlight the areas that need attention, particularly during the pandemic. According to the present results from the service provider perspective, two categories of COVID-related services and the non-COVID-related ones can be important during the pandemic. According to a cohort study in Portugal, the older patients have a twice larger need for admission in the ICUs than other age groups [56].

According to the present results, access to home care services, telehealth, oral and mental healthcare, palliative healthcare, medication and routine healthcare are among the significant factors in non COVID-related services for the elderly. In this regard, other evidence shows that the COVID-19 condition can change the health care systems by reducing the need for face to face visits and the limitations in appropriate palliative services for those who suffer from cancers and their medications [57]. At the same time, as Banerjee (2020) stated, the fear and uncertainty resulting from the pandemic can cause older people to suffer more senses of loss, anxiety, fear, loneliness, or sometimes, self-neglect and indifference [58]. This can clarify the need for particular attention to access mental health services during the pandemic, especially among the elderly.

Another important sub-theme, the access to telehealthcare along with digital literacy, is considerably emphasized in the present study. The pandemic condition requires alternative facilities aiming to replace traditional care with telehealth. These changes are obvious in the areas of consulting, oral health, palliative care and so on. Nonetheless, the use of telehealth services among the elderly can raise various concerns of their lack of digital literacy, increasing misinformation and lack of confidence or the ability to use the technology. In this regard, evidence shows that some older people’s demographic characteristics, together with visual and auditory abilities, and their physical and mental capabilities, can highly affect their tendency and ability to accept and use tele health care [59]. These items and their digital literacy and education level and the need for developing the applications and devices so that both the older people and their caregivers can benefit them are among the considerable recommendations in this area.

Apart from the aforementioned micro and personal factors, other present findings have emphasized the role of health policies and the system’s structure along with a complex of demographic, physical, social, cultural and economic factors. For example, Doetsch et al. (2017) have proposed that health policies such as health reforms, allocated health budgets and the degree of communication between different levels of the health sector have a positive association with equality in access to healthcare services among the elderly [60].

Saeed et al. (2016) have also confirmed that health status, income, education, health insurance, employment and residence status are among socio-economic factors that can affect the utilization of healthcare services by the elderly [61]. Furthermore, according to Qureshi (2002), demographic factors and social and economic determinants can affect the political directions and the provision of health services for the elderly and the number of allocated resources to the older population’s health needs [62]. Hamiduzzaman et al. (2017) have also noted that some factors like overall health status, healthcare needs, social and economic factors and cultural determinants can more affect the access to healthcare services by the elderly than existing healthcare centers and facilities [63].

### Limitations

The inability to access the full-text document of all the abstracts potentially fulfilling the inclusion criteria should be considered as one of the limitations of the present study. Another limitation can be the potentially restricted number of original articles or reviews in this area due to the short time from the beginning of the pandemic.

## Conclusions

Results of this study have shown that healthcare provider-level factors can affect the access to health care services for the elderly during the pandemic. These determinants include access to health services both related to the diagnosis and treatment of COVID-19 and the routine services non-related to COVID-19. Furthermore, some micro factors at the personal level can influence the elderly’ utilization of health services, such as accommodation challenges, the perceived attitude of aging and the level of literacy and education of the elderly. Also, the macro determinants, which are the health policies and the system’s structure and a complex of demographic, physical, social, cultural and economic factors, are considered important in this area. Considering all these factors together can shed light for policymakers to achieve a broader view of the issue and, as a result, aim to follow a new direction to seek better and more equitable interventions and decisions for the elderly’s access and utilization of healthcare services during the COVID − 19 pandemic.

## Supplementary Information

**Additional file 1.** Table A-Supplement- The characteristics of the included studies.

## Data Availability

While identifying/confidential patient data should not be published within the manuscript, the datasets used and/or analyzed during the current study are available from the corresponding author on reasonable request.

## Abbreviations

**ICU**: Intensive Care Units
**QDA**: Qualitative Document Analysis
