# Supplementary material for: Factors influencing access and utilization of health services among older people during the COVID − 19 pandemic: a scoping review
Source: Arch Public Health. 2021 Nov 7;79:190. doi: 10.1186/s13690-021-00719-9 (PMC8572573; doi:10.1186/s13690-021-00719-9)
Supplement: Supplementary file 1 — Additional file 1. Table A-Supplement- The characteristics of the included studies. [file 13690_2021_719_MOESM1_ESM.docx]

| Table A-Supplement- The characteristics of the included studies | | | | |
| --- | --- | --- | --- | --- |
| row | title | first author | country | design |
| 1 | COVID 19 - Clinical Picture in the Elderly Population: | Agnieszka Neumann Podczaska | Poland. | review |
| 2 | Equity and elderly health in India: reflections from 75th round National Sample Survey, 2017–18, amidst the COVID-19 pandemic | Alok Ranjan | India | Cross Sectional |
| 3 | COVID-19 lockdown impact on lifestyle habits of Italian adults | Anna Odone | Italy | COMMENTARY |
| 4 | COVID-19: the implications for suicide in older adults | ANNE PAMELA FRANCES WAND | china | COMMENTARY |
| 5 | Effect of the COVID-19 lockdown on disease recognition and utilization of healthcare services in the older population in German: a cross-sectional study | Bernhard Michalowsky | German: | Cross Sectional |
| 6 | The Unique Impact of COVID-19 on Older Adults in Rural Areas | Carrie Henning-Smith | U.S. | COMMENTARY |
| 7 | COVID-19: The forgotten priorities of the pandemic | Cristina Mesa Vieiraa | Colombia | V I E WPOINT |
| 8 | Community Susceptibility and Resiliency to COVID-19 Across the Rural-Urban Continuum in the United States | David J. Peters, | United States | Cross Sectional |
| 9 | Medical Home Visit Programs During COVID-19 State of Emergency | Donna Seminara | United States | COMMENTARY |
| 10 | Telehealth for the cognitively impaired older adult and their caregivers: lessons from a coordinated approach | Erica F Weiss | United States | Report |
| 11 | Impact of COVID‑19 pandemic on rheumatoid arthritis from a Multi‑Centre patient‑reported questionnaire survey | Esam Abualfadl | Egypt | Cross Sectional |
| 12 | The Effects of COVID-19 Among the Elderly Population: A Case for Closing the Digital Divide | Gabrielle Martins Van Jaarsveld | Netherlands | VIEWPOINT |
| 13 | Aged Patients With Mental Disorders in the COVID-19 Era: The Experience of Northern Italy | Gianluca Serafini, | Italy | Letter to the Editor |
| 14 | Strategies to promote access to medications during the COVID-19 pandemic | J Simon Bell | Australia | COMMENTARY |
| 15 | Hospital care for elderly COVID-19 patients* | Jack Roberto Silva Fhon | Brazil | document analysis |
| 16 | After the COVID-19 Pandemic: The Next Wave of Health Challenges for Older Adults | Jennifer A. Schrack | United States | Letter to the Editor |
| 17 | Compliance to Recommendations and Mental Health Consequences among Elderly in Sweden during the Initial Phase of the COVID-19 Pandemic | Johanna Gustavsson | Sweden | Cross Sectional |
| 18 | COVID-19 in older adults: clinical, psychosocial, and public health considerations | John P. Mills | United States | V I E WPOINT |
| 19 | The Interpersonal and Psychological Impacts of COVID-19 on Risk for Late-Life Suicide | Julia L. Sheffler, | United States | COMMENTARY |
| 20 | Older Adults with COVID-19 Can Choose Care at Home: Lessons Learned from New York City | JustinaL.Groeger | United States | COMMENTARY |
| 21 | Acute, Post-acute, and Primary Care Utilization in a Home-Based Primary Care Program During COVID-19 | Karen A. Abrashkin | United States | quasi-experimental study |
| 22 | The COVID-19 Pandemic Exposes Limited Understanding of Ageism | Laurinda Reynolds M.A. | United States | essay |
| 23 | SARS-CoV-2 pandemic and the population with dementia. Recommendations under the auspices of the Polish Psychiatric Association | Mateusz Łuc1 | Poland | COMMENTARY |
| 24 | “Will you hear my voice?”: to engage older patients online, listen to them about their lives offline | Michael L. Millenson | Israel | COMMENTARY |
| 25 | ‘An invisible human rights crisis’: The marginalization of older adults during the COVID-19 pandemic | Migita D'cruz | India | review |
| 26 | Reconnecting: Strategies for Supporting Isolated Older Adults during COVID-19 through Tele palliative Care | Regina M. Mackey | United States | V I E WPOINT |
| 27 | Study of COVID-19 Pandemic in Representative Dialysis Population Across Mumbai, India: An Observational Multicentric Analysis | Rushi Deshpande | India | Cross Sectional |
| 28 | Older Adults, Social Technologies, and the Coronavirus Pandemic: Challenges, Strengths, and Strategies for Support | Ryan C. Moore | United States | V I E WPOINT |
| 29 | COVID-19 and Inequities in Oral Health Care for Older People: An Opportunity for Emerging Paradigms | S. León | china | V I E WPOINT |
| 30 | Impact of COVID-19 on loneliness, mental health, and health service utilization: | Samuel Yeung Shan Wong | Hong Kong | prospective cohort study |
| 31 | Caring for Older Adults With Diabetes During the COVID-19 Pandemic | Sarah L. Sy, | United States | VIEWPOINT |
| 32 | Older Adults and Covid-19: The Most Vulnerable, the Hardest Hit | TIA POWELL | United States | VIEWPOINT |
| 33 | COVID-19 and older adults in Africa: Social workers’ utilization of mass media in enforcing policy change | Tracy BE Omorogiuwa | Nigeria | Brief Note |
| 34 | COVID-19 pandemic and mental health care of older adults in India | VIHANG N. VAHIA1 | India | COMMENTARY |
| 35 | Mental health services for older adults in China during the COVID-19 outbreak | Yuan Yang | china | Correspondence |
| 36 | Access to healthcare for people aged 50+ in Europe during the COVID‑19 outbreak | Šime Smolić | European countries | Cross Sectional |
| 37 | The effect of COVID-19 pandemic and lockdown on consultation numbers, consultation reasons and performed services in primary care: results of a longitudinal observational study | Ingmar Schäfer | Germany | Cross Sectional |
| 38 | Factors associated with virtual care access in older adults: a cross-sectional study | Laura Liu | Canada | Cross Sectional |
| 39 | Unequal Impact of Structural Health Determinants and Comorbidity on COVID-19 Severity and Lethality in Older Mexican Adults: Considerations Beyond Chronological Aging | Omar Yaxmehen Bello-Chavolla | Mexico | Cross Sectional |
| 40 | Acute, Post-Acute, and Primary Care Utilization in a Home-Based Primary Care Program during COVID-19 | Karen A. Abrashkin | USA | Cross Sectional |
| 41 | A UK survey of COVID‐19 related social support closures and their effects on older people, people with dementia, and carers | Clarissa Giebel | UK | Cross Sectional |
| 42 | Primary healthcare utilisation by older Australians during the COVID-19 pandemic | Jonathan D. Bartholomaeus | Australia | Letter to editor |
| 43 | Change of Use in Community Services among Disabled Older Adults during COVID-19 in Japan | **Tomoko Ito** | Japan | Cross Sectional |
| 44 | Barriers to telehealth access among homebound older adults | Alexander V. Kalicki BS | USA | Cross-sectional |
| 45 | A qualitative study on the elderly and accessibility to health services during the COVID-19 lockdown in Buenos Aires, Argentina | Candela Agustina Loza | Argentina | qualitative |
| 46 | Older adults with non-communicable chronic conditions and their health care access amid COVID-19 pandemic in Bangladesh: Findings from a cross-sectional study | **Sabuj Kanti Mistry** | Bangladesh | Cross-sectional |
| 47 | Accessibility of Telehealth Services During the COVID-19 Pandemic: A Cross-Sectional Survey of Medicare Beneficiaries | Boon Peng Ng | USA | Cross-sectional |
| 48 | Epidemiology, outcomes, and utilization of intensive care unit resources for critically ill COVID-19 patients in Libya: A prospective multi-center cohort study | **Muhammed Elhadi** | Libya, | Cohort |
| 49 | Palliative Care for Older Adults with Multimorbidity in the Time of COVID 19 | Victoria D. Powell | USA | review |
| 50 | A national survey on COVID-19 second wave lockdowns on older adults’ mental  wellbeing, health-seeking behaviours and social outcomes across Australia | **Joyce Siette** | Australia | Cross-sectional |
